# Will Dengue Vaccines Be Used in the Public Sector and if so, How? Findings from an 8-country Survey of Policymakers and Opinion Leaders

**DOI:** 10.1371/journal.pntd.0002127

**Published:** 2013-03-14

**Authors:** Don L. Douglas, Denise A. DeRoeck, Richard T. Mahoney, Ole Wichmann

**Affiliations:** 1 DKT Janani, Reshmi Complex, P&T Colony, Kidwaipuri, Patna, India; 2 International Vaccine Institute, Kwanak-gu, Seoul, South Korea; 3 Robert Koch Institute, Immunization Unit, DGZ-Ring1, Berlin, Germany; The George Washington University Medical Center, United States of America

## Abstract

**Background:**

A face-to-face survey of 158 policymakers and other influential professionals was conducted in eight dengue-endemic countries in Asia (India, Sri Lanka, Thailand, Vietnam) and Latin America (Brazil, Colombia, Mexico, Nicaragua) to provide an indication of the potential demand for dengue vaccination in endemic countries, and to anticipate their research and other requirements in order to make decisions about the introduction of dengue vaccines. The study took place in anticipation of the licensure of the first dengue vaccine in the next several years.

**Methods/Principal Findings:**

Semi-structured interviews were conducted on an individual or small group basis with government health officials, research scientists, medical association officers, vaccine producers, local-level health authorities, and others considered to have a role in influencing decisions about dengue control and vaccines. Most informants across countries considered dengue a priority disease and expressed interest in the public sector use of dengue vaccines, with a major driver being the political pressure from the public and the medical community to control the disease. There was interest in a vaccine that protects children as young as possible and that can fit into existing childhood immunization schedules. Dengue vaccination in most countries surveyed will likely be targeted to high-risk areas and begin with routine immunization of infants and young children, followed by catch-up campaigns for older age groups, as funding permits. Key data requirements for decision-making were additional local dengue surveillance data, vaccine cost-effectiveness estimates, post-marketing safety surveillance data and, in some countries vaccine safety and immunogenicity data in the local population.

**Conclusions/Significance:**

The lookout for the public sector use of dengue vaccines in the eight countries appears quite favorable. Major determinants of whether and when countries will introduce dengue vaccines include whether WHO recommends the vaccines, their price, the availability of external financing for lower income countries, and whether they can be incorporated into countries' routine immunization schedules.

## Author Summary

Information gleaned from surveys of country-level policymakers and other opinion leaders can assist in planning the development, production and introduction of new or upcoming vaccines into public sector immunization programs. In the case of dengue vaccines, prevailing views among these leaders about the importance of the disease, their expressed level of interest in the government's use of the vaccine, and preferred strategies for vaccine introduction (e.g., geographically-targeted vs. nation-wide vaccination, specific age groups to target) can help to identify “early adopter” countries and indicate the level of demand for the vaccine. This information can be critical to current producers of the vaccine in planning their production capacity and to potential future producers in deciding whether to pursue development of the vaccine. This information also helps donors and international technical agencies, such as WHO and UNICEF, in setting their priorities and determining their level of technical and financial support to countries for the introduction of dengue vaccines. In addition, these surveys can provide crucial information to national governments and the above stakeholders about potential barriers to introducing dengue vaccines into national immunization programs, and what additional studies and data countries will require in order to make decisions about use of the vaccines in the public sector.

## Introduction

Dengue, a mosquito-borne Flavivirus infection caused by four related viruses (DENV1 to 4), is a major public health problem in the tropics and subtropics. The greatest documented burden of dengue occurs in Asia and Latin America. Dengue's geographic range now places an estimated 3.97 billion people at risk and it continues to expand, causing epidemics that disrupt health care systems [1], [2]. The World Health Organization estimates that each year there may be up to 50 million dengue infections worldwide and 500,000 cases of dengue hemorrhagic fever (DHF) – a severe form of the disease [3]. According to the 2010 Global Disease Burden Study, dengue causes nearly 15,000 deaths per year, a 29% increase since 1990 [4].

Dengue vaccines have been under development since the 1940s, but the vaccine industry's interest in the vaccines languished throughout most of the 20^th^ century [5]. However, dengue vaccine development has accelerated in recent years and several vaccine candidates are in or near to human clinical development. The most advanced is a recombinant live chimeric tetravalent vaccine (CYD TDV) developed by Sanofi Pasteur [6], consisting of four genetically engineered viruses in which several genes in a yellow fever DNA backbone have been replaced with comparable genes from the four dengue viruses. The vaccine is being evaluated in a three-dose regimen given over a one-year period (at six month intervals) in efficacy trials in multiple countries in Asia and Latin America. Although this and all other dengue vaccines are intended to be used with children and adults, initial licensure is likely to be limited to children 2–14 years of age, since the current clinical trials are evaluating the vaccine in this age group.

Two other live attenuated chimeric dengue vaccine candidates in are Phase II evaluations: one developed by the U.S. National Institutes of Health [7], and another being developed by Inviragen [8]. In addition, a recombinant subunit vaccine under development by Merck has completed Phase I trials [9].

This survey of eight dengue-endemic countries in Asia and the Americas was conducted by the Pediatric Dengue Vaccine Initiative (PDVI), managed by the International Vaccine Institute, to determine their possible interest in and perceived need for a dengue vaccine; what factors (e.g., vaccine characteristics, financing, political pressure, data needs) would drive or influence decisions; and what strategies countries would likely use to target, deliver and finance dengue vaccination. The main objectives of the survey were to: 1) provide an indication to donors, vaccine producers, and the international health community of the potential interest in and demand for dengue vaccination from endemic countries and what factors may affect this demand; and 2) anticipate the research, disease surveillance and other requirements that countries will have in order to make decisions about the introduction of dengue vaccines.

Surveys of policymakers and other stakeholders about new or under-utilized vaccines have been used in the past to inform research and advocacy activities of product development partnerships, including a multi-country study about rotavirus vaccines [10] and a seven-country survey concerning cholera, typhoid fever and shigellosis and vaccines against these diseases [11].

## Methods

### Country selection

The survey was conducted in four Asian countries (India, Sri Lanka, Thailand and Vietnam) and four Latin American countries (Brazil, Colombia, Mexico and Nicaragua). This was a convenience sample of countries that are considered dengue-endemic where PDVI had contacts viewed as reliable authorities on dengue in their countries and who could facilitate arrangements for interviews with policymakers. Several of the countries were viewed by the investigators as potential early adopters of dengue vaccines based on reports of dengue outbreaks and expressed concern from health officials and the public about dengue in the country. No country that was requested to participate in the study refused to do so.

### Data collection and selection of topics

The study consisted of face-to-face interviews conducted on an individual or small group basis during country visits that took place between September 2008 and December 2010. All policymakers and other stakeholders interviewed consented to the interviews, which were voluntary, and participants were informed that their responses would be anonymous.

The interviews were semi-structured, using a question guide consisting entirely of open-ended questions (Appendix 1). The guide explored informants' views and perceptions about:

- the magnitude and seriousness of dengue in their country, as well as epidemiological patterns and trends;
- the level of priority of dengue control;
- the quality and accuracy of dengue surveillance data;
- the effectiveness of current dengue prevention and treatment methods;
- the need for and interest in dengue vaccines;
- preferred attributes and criteria for dengue vaccines;
- required data to inform decisions about the introduction of dengue vaccines; and
- strategies for vaccine introduction and use (including age and geographical targeting, vaccine delivery methods and channels, and financing strategies).

The semi-structured interview approach was felt to be the most appropriate for high-level informants, as opposed to a highly-structured questionnaire. This format facilitated the free expression of opinions and ideas among informants, allowed for probing and clarification of responses, and for the identification of new issues and topics as they arose. All informants were asked a core set of questions concerning their views about dengue, current prevent and control measures, and various aspects about dengue vaccines, while additional specific questions were asked to individuals, according to their expertise and position. For example, immunization program officials were asked about government plans and priorities for new vaccine introductions and vaccine producers were asked about the status and plans of vaccine development.

The interviews were conducted in English by one or more of the authors in each country. Local collaborators who arranged the interviews and meetings, also sat in on several of them and served as translators, as needed, in interviews conducted in the Latin American countries.

### Selection of interviewees (“informants”)

Persons to be interviewed in each country were identified based on a list developed by PDVI of the types of organizations and individuals to target. An effort was made to meet as many individuals and groups as possible who potentially have a role in making or influencing decisions about the future introduction of dengue vaccines into national immunization programs, including decision-makers within health ministries; chairs or members of national immunization technical advisory groups (NITAGs); immunization program heads; international aid agencies; leading scientists; national regulatory authorities; and local vaccine producers. Efforts were also made to meet with the highest level officials in each organization and category as possible. If people who were suggested to be interviewed were not available or did not respond, they or their office usually recommended a colleague within the organization or department to interview.

As shown in Table 1, those interviewed included senior officials from ministries of health (MOHs) (e.g., Directors General of Health Services, health secretaries and directors of communicable diseases) and other government health agencies (e.g., centers for disease control, national regulatory authorities, national health insurance agencies). They also included national immunization program managers (in five countries); and MOH officials and scientists from dengue control and vector control programs, disease surveillance and epidemiology, infectious disease control and planning departments. Other informants included officials and scientists from public and private research institutes and academic institutions; top officials of major hospitals; officers of medical associations; health authorities at the regional, state and/or municipal level (including state or provincial health ministers or the equivalent and chief medical officers); officials from international technical agencies (e.g., WHO, UNICEF); and representatives from local vaccine producers (in India, Vietnam, Brazil and Mexico) or multi-national pharmaceutical companies (in Colombia). Several of those interviewed also served on their country's NITAG.

**Table 1:** Numbers of persons who took part in interviews or meetings by country and type of position.

Type of Position	India	Sri Lanka	Thailand	Vietnam	Brazil	Colombia	Mexico	Nicaragua	Total
Senior government policymakers, program managers, department heads and other officials in health ministries and other health agencies	10	12	6	10	4	6	6	7	61
Hospital directors and other representatives	2	2	5	5	1	–	1	5	21
Scientists from research institutes and academia	2	1	1	2	7	2	2	1	18
Officers from professional associations^a^	2	–	–	–	1	3	2^c^	3	10
Local-level health policymakers and other officials (state, provincial, municipality)	1	4	–	–	1	–	3	4	13
Representatives from local vaccine producers and multi-national pharmaceutical companies^b^	8	–	–	1	6	2	1	–	18
International agency officials (WHO, PAHO, UNICEF)	7	–	1	3	–	1	–	1	13
National regulatory agency/laboratory officials	–	–	1	–	1	–	–	1	3
Other	–	–	–	–	–	–	1	–	1
Total number of informants	32	19	14	21	21	14	15	22	158

a Includes Presidents, General Secretaries and other top officials from national medical, pediatric, infectious disease and public health associations.

b All were from local vaccine producers, except in Colombia, where representatives from Sanofi Pasteur were interviewed.

c One of the professional society officials in Mexico was counted in another category and thus not included in the total.

Between 14 and 32 persons took part in the interviews in each country, for a total of 158 individuals (average of ≈20 per country).

### Data analysis

The results were analyzed separately for each country. Extensive notes were taken for each interview and a complete set of notes were transcribed by topic area and then by person or group interviewed, including salient quotations. From these transcripts, the types and patterns of responses were analyzed by the type and level of informants. Responses were reported if at least two persons in a country gave a similar response. Individual country reports were then prepared that included sections on perceptions about the importance and priority of dengue in the country; perceptions about current dengue prevention and control; the perceived need for dengue vaccines; concerns, criteria and data needs regarding dengue vaccines; and possible vaccine introduction strategies and scenarios. Both the raw data (interview notes) and country reports were used in preparing this manuscript.

## Results

### Perceived importance and priority of dengue

The majority of policymakers, hospital directors, and other informants whose work is not focused solely on dengue considered dengue an important disease and a priority in all eight countries included in the study. The disease was called a “major public health problem” by a senior health official in India, and “right at the top” [of health priorities] by a senior MOH official in Sri Lanka. Dengue was deemed “unappreciated by donors and the government” by preventive medicine officials and NITAG members in Vietnam. Similarly, senior health policymakers in Colombia described the disease as a “very high priority” and “very important” in terms of hospitalization and loss of productivity. In Brazil, the chair of the country's NITAG described dengue as one of only two diseases without effective control (the other being leishmaniasis) and considered it a high priority, along with tuberculosis, hepatitis and HIV/AIDS. In Nicaragua, dengue was described by a senior Ministry of Health official as not normally one of the top 20 priority diseases, but when there is an outbreak, it rises to the top and becomes the number one priority.

The disease is especially a high priority among those on the frontlines of dengue prevention and control. City health officials in Colombo, Sri Lanka ranked dengue their number one disease priority, and it is the only disease which the city government tracks through GIS mapping. City health officials also keep a map showing all cases of the disease on a large poster board (along with leptospirosis) that is updated daily.

Agreement among policymakers and other informants about the importance of dengue was not universal, however. A senior health official in India did not consider dengue a top priority like malaria, while some hospital officials in the country believe that the extensive media attention given to dengue outbreaks takes the focus away from other important endemic diseases, such as diarrhea, enteric fevers and hepatitis. And according to some hospital officials in Thailand, dengue is now less recognized by politicians as a major problem due to the country's success in reducing dengue shock syndrome (DSS) and deaths to a low level.

The main reasons why informants considered dengue a priority disease are the following:

- **The disease is spreading within countries and is no longer confined to cities**

This was a key factor mentioned by informants in all countries except Colombia and Nicaragua (where dengue is still largely considered an urban disease). According to interviewees in Sri Lanka, for instance, dengue was mainly confined to the city of Colombo in 1996, but by 2004 it had spread to 10 districts, and by 2007 to nearly all of the country's 26 districts. In Vietnam, where dengue has been endemic in the South for decades, outbreaks occurred in the North of the country in 2008 for the first time. The spread of the disease to peri-urban and even rural areas was a common perception and concern among informants in India, Sri Lanka, Vietnam, Thailand, Brazil, and Mexico, which they attribute to increased development of these areas, leading to building construction, proliferation of garbage, open water storage and other conditions favorable to the breeding of dengue-carrying mosquitoes. According to one informant in Brazil, 40% of dengue cases reported since 2007 have come from the outskirts of large cities. Some informants in Vietnam, Thailand and India believed that dengue is considerably more under-reported in rural areas than in cities and that the incidence could be as great in rural areas as in urban areas.

- **Growing incidence and increased frequency of major outbreaks**

Informants in several countries, including Sri Lanka, Thailand and Nicaragua, report an increase in dengue incidence over the past several years. Those in the Asian countries of Sri Lanka, Vietnam, Thailand report that major outbreaks are now occurring every year, while as recently as five or six years earlier, they used to occur every three or four years, The disease was described in Sri Lanka as changing from an epidemic disease to a “hyper-endemic” disease that occurs throughout the year.

- **The epidemic pattern of the disease, overwhelming hospitals and causing fear and panic in the population**

According to dengue researchers in Brazil, dengue overwhelms the health care system in urban areas during outbreaks more than any other disease, in terms of its numbers and severity. Informants in other countries report a similar phenomenon. During a major outbreak in Delhi, India in 2006, dengue wards were set up in tents outside of a major hospital, where 1,200 cases were treated daily. Dengue cases make up thirty to forty percent of all pediatric patients at a provincial hospital in Thailand during outbreaks. And at a national children's hospital in Managua, Nicaragua, the number of beds was increased 64% during an outbreak in 2009, mainly to accommodate dengue patients. The burden placed on hospitals during dengue outbreaks is increased even more by the panic that strikes the public, according to informants in Sri Lanka and Brazil. One expert in Sri Lanka describe “dengue phobia”, which causes parents to rush their child to a health facility at the first sign of a fever, fearing that it's dengue.

- **The highly visible and political nature of the disease**

Because dengue occurs in outbreaks and urban populations are affected, it garners considerable media attention, placing pressure on politicians – especially at the local level – to find a way of controlling the disease. According to one informant in Thailand, whenever a child dies of dengue, it is reported in the media. Given this visibility, there can be high costs to political leaders viewed as failing to control outbreaks. In Brazil, mayors as well as officials responsible for vector control have been replaced because of dengue outbreaks, according to informants. Outbreaks in Colombia have prompted political leaders to declare a state of emergency, as occurred in Cali in 2010 after more than 1,200 cases and nine deaths were reported.

- **The rapid onset of severe symptoms**

Informants in both Asian and Latin American countries (India, Thailand, Nicaragua) gave as a major reason for dengue being a priority the fear among physicians of an otherwise healthy, well-nourished child deteriorating in a matter of hours, leading to DSS, other complications, and even death. As one hospital director in Thailand stated, “Death due to dengue is particularly tragic, because children go from healthy to fatal very rapidly.” The speed at which complications developed during a major dengue outbreak in Managua, Nicaragua in 2008 took medical experts and hospital officials by surprise; even within the first day of the onset of symptoms, patients were developing severe symptoms, including circulatory shock. However, informants in Thailand and Vietnam pointed out that rates of DSS and dengue-associated deaths have remained the same or have even decreased in the past decade or so, due to early and more effective treatment.

- **Shifts in the age patterns of the disease**

Dengue has been viewed as mainly a children's disease in Asia and as an adult disease in Latin America. However, in some Latin American countries, notably Brazil, children are increasingly being affected by the disease, causing concern in the medical and public health community. A dramatic shift in DHF cases from adults to children occurred in Brazil in 2007 [12], which informants claim is responsible for the increased severity of the disease in the country.

- **The economic impact of dengue on government budgets**

Informants interviewed in all countries except India gave the economic impact of dengue as a reason for their concern about the disease. In Colombo, Sri Lanka, 20% of the city government's entire health budget – which covers the cost of health clinics, maternity homes, water quality and many other activities – is spent on dengue-related activities. Officials in one province in Nicaragua estimated that dengue control efforts during outbreaks typically consume 60% of the province's emergency budget. Hospital costs during dengue outbreaks can also put a severe strain on health budgets, as reported in Mexico.

- **Other factors**

Another reason given in some countries for the perceived growing importance of dengue is the great progress made in controlling or reducing mortality from other major infectious diseases at the same time as dengue incidence is increasing and/or expanding geographically. In Sri Lanka, the control of tuberculosis and malaria through intensive treatment, Japanese encephalitis (JE) through vaccination, and diarrheal disease deaths through oral rehydration therapy has reduced the relative importance of these diseases among policymakers, resulting in dengue rising to the top of infectious disease priorities. Similarly, in Nicaragua, informants pointed out that malaria and several other diseases (measles, rubella) have largely been brought under control in recent years, while little progress has been made on dengue.

A further reason for the sense of priority of dengue among informants, as mentioned in Thailand, Brazil and Nicaragua, is the fact that the disease strikes all sectors of society – rich and poor alike – and thus no one is immune from getting the disease.

### The level of interest in dengue vaccines among policymakers and opinion leaders

Interest among policymakers and opinion leaders in the public sector use of dengue vaccine was on the whole quite high – though not universal – in the eight countries surveyed. A senior MOH official in Sri Lanka, where interest in dengue vaccines was universally high amongst those interviewed, believed that the government would introduce a dengue vaccine if it was affordable, even in the absence of donor funding. Similarly, a health policy expert in Mexico claimed that a dengue vaccine would be accorded a “high priority” by the government once one becomes available. And according to a high-level health ministry official in Nicaragua, the government would have a “genuine interest in making a [dengue] vaccine available to the population that needs it most.” A provincial health official in the country claimed that the introduction of a dengue vaccine would be “a priceless achievement in public health”, saving the country, the health system and people money.

Government officials in India were more hesitant to state an interest by the government in using dengue vaccines, in the absence of data about their safety and performance. A senior health official was skeptical of the need for the vaccine, claiming that vector control is preferable to a vaccine and is succeeding. However, expressed interest in dengue vaccines in India was high among non-government informants on the frontline of treating dengue cases, such as hospital officials and representatives of professional medical associations.

According to interviewees in several countries (Sri Lanka, India, Thailand, Brazil, Mexico), a key driver of government interest in dengue vaccines is the great pressure that the public and in some cases, the media will put on the government to introduce the vaccines, once available. The high expected public demand is due to the outbreak pattern of the disease, the media attention that it attracts, the public's high awareness of the disease in these countries, and the fear that it engenders in the population. In Thailand, interviewees believed that such pressure would result from the inequities created by having a dengue vaccine available in the private market but not through the national immunization program for free. Pressure would also come from the medical community in many countries, given the lack of specific treatment for dengue and the difficulty in predicting its course. According to several informants in Sri Lanka, such population demand, coupled with media pressure, is a stronger driver of new vaccine introductions than evidence of disease burden or cost-effectiveness, since politicians “are sensitive to population demand”. An official from an Indian vaccine producer, in fact, described vaccines against dengue and Japanese encephalitis (JE) as “political vaccines”, given the public's tendency to blame politicians for outbreaks of these vector-borne diseases in their community. The potential political benefits of introducing a dengue vaccine is described by a health policy expert in Mexico: “A courageous and early decision taken about dengue vaccines by the next government could be an early win for [them]. Launching a dengue vaccine to coincide with the political cycle could be most helpful.”

Despite the generally high level of interest in dengue vaccines expressed by most informants in the eight countries, several, including government officials in India, Thailand, Mexico and Nicaragua, were more cautious about embracing vaccines whose safety, performance and cost are not yet known. According to these informants, questions about a dengue vaccine's safety, the number of doses required, its effectiveness and duration of protection, as well as its affordability and financing, would have to be answered before their government would use it in the public sector. As one provincial government official from Nicaragua stated, “It is important not to raise unrealistic public expectations about dengue vaccines and their ability to stop infections soon after introduction.” The relatively low mortality of the disease could also be an obstacle to its rapid introduction into government immunization programs, according to some informants. As one informant in Mexico declared, “The government would need to be convinced of the importance of making large budget allocations for dengue vaccines, since there are only around 2,000 cases of DHF and 20 deaths per year.”

### Concerns, criteria and preferences regarding dengue vaccines

The top concerns that informants had about dengue vaccines were their safety, cost and whether they can be used in infancy or early childhood.

#### Safety

Safety concerns centered around three main issues:

1. Whether the vaccine can lead to enhanced disease since previous dengue infections increase the risk of severe disease from subsequent infections with different dengue serotypes [13]. Informants were concerned that such cross-enhancement could develop in people exposed to the disease before they receive all vaccine doses or if the vaccine does not confer long-lasting protective antibodies against all four dengue serotypes;
2. Whether there will be interference between dengue and JE antibodies induced by either natural JE infection or by JE vaccination (raised by informants in Sri Lanka and Thailand);
3. Whether live vaccines could convert to virulence or could introduce a new serotype into a country.

#### Vaccine cost

According to those interviewed, the price of dengue vaccines to the public sector would be a major determinant of whether or not governments would introduce the vaccine (as mentioned in India and Colombia), how widespread its introduction would be (Mexico, Vietnam), or whether introduction would be delayed (Thailand). Most informants would not give a maximum acceptable price of the vaccine for the public sector, but those that did gave thresholds reflective of their country's income level. A Thai official suggested that a price of less than $10 a dose would be acceptable, while the maximum acceptable price given by Mexican informants ranged from less than $10 to $15 per dose. A university health economist in Colombia who conducts economic analyses of new vaccines felt that full introduction would take a long time unless the vaccine costs no more than $5.00 per dose. In the much poorer country of Vietnam, one MOH official believed that $5 per dose was much too expensive, while another gave a preferred price of $1 per dose.

#### Minimum age of effectiveness and vaccination schedule

There was a strong preference expressed among informants in all countries for a dengue vaccine that protects children as young as possible – that is before they are exposed to any of the four dengue viruses. Policymakers also strongly preferred a vaccine that would fit into their existing routine child immunization schedule. When asked about the acceptability of a vaccine that cannot be given until the age of one – the likely minimum age at which live attenuated dengue vaccines can be given due to interference from circulating maternal antibodies in infants – informants in most countries considered a schedule that starts at 12 months of age (or 9–12 months) acceptable. This is in part because many countries in the study have extended their immunization schedule to the second year of life and beyond, as they introduce new vaccines not given to infants (e.g., JE, human papillomavirus (HPV)), and add booster doses of other routine vaccines (e.g., DTP, OPV) to the schedule. In Sri Lanka, for example, there are now five contacts in the immunization schedule from the age of one to school entry.

A dengue vaccine requiring two doses was viewed as acceptable by most informants across countries. However, a three-dose regimen was considered a problem in India – because of significant dropout rates in the country – as well as in Brazil. Few interviewees would state specific thresholds of acceptable efficacy rates for a dengue vaccine. Some in Vietnam and India felt that the vaccine should be at least 90% protective, while others in Vietnam would accept rates as low as 80–85% or even 70%. A senior academic and NITAG member in Sri Lanka would accept a vaccine that is at least 50% efficacious.

#### Other criteria

A WHO recommendation for the use of dengue vaccines was cited as an important criterion for introduction into public sector programs by several informants in Vietnam, Thailand and India. According to interviewees in Nicaragua and Colombia, WHO pre-qualification of the vaccine would also be required before it could be introduced into their national immunization program.

### Possible strategies for dengue vaccine introduction

#### Scope of vaccination

Informants in Sri Lanka and Thailand believed that dengue vaccine introduction in their countries would be nation-wide, since the disease is considered endemic throughout both countries. However, it could be rolled out in a few provinces initially to demonstrate the vaccine's effectiveness, determine the best strategies for implementation, and work out logistical challenges. There was also preference for nation-wide introduction of the vaccine among informants in Nicaragua.

However, in the remaining five countries, most informants discussed targeting high-risk areas initially, with the possibility of eventual introduction nation-wide, if funding is available, the price of the vaccine – assumed to be high initially – is reduced to affordable levels, and the vaccine supply is sufficient. According to some interviewees in Mexico, if dengue vaccines are initially very expensive, it could still be introduced quite early after its entry into the market, but on a very limited basis municipality by municipality, based on an analysis of disease incidence. Some Mexican informants would also prioritize tourist areas, such as Monterey, Cancun and Acapulco, to protect the “national image” and the important tourism industry.

In both Mexico and Colombia, informants raised the possibility of local governments introducing and financing the vaccine on their own before it is adopted by the national immunization program. There is precedent for this in Colombia, where the city of Bogota had purchased some new vaccines, including pneumococcal conjugate and hepatitis A, that were not yet available through the national immunization program at the time of the survey.

In India – where dengue is now considered endemic in 18 out of the 35 states – two scenarios were proposed by various informants, both beginning with the use of the vaccine in the private sector. In one scenario, certain municipality and state governments would introduce dengue vaccine with their own funding, due to the high political costs that dengue outbreaks can exact on local politicians. The other scenario would follow the model of JE vaccine introduction: outbreaks would occur, creating outrage from the public once they learn that a vaccine exists, leading affected states to demand that the Universal Immunization Program (UIP) provide the vaccine in high-risk areas.

#### Target ages and delivery strategies

Informants in all countries believed that children would be the top priority for dengue vaccination, beginning with the youngest ages eligible to receive the vaccine, preferably through the routine immunization schedule. As with geographic targeting, several countries would phase in vaccination to other age groups, as the vaccine price decreases and funding allows. According to informants in Brazil, Mexico, Colombia and Thailand, pre-school and school-aged children up to the age of 15 would next be targeted, likely through school-based catch-up campaigns.

Expanding government-financed dengue vaccination to adults was specifically mentioned only in Brazil, Colombia, and Mexico – three Latin American countries where adults still account for a substantial portion of the dengue disease burden. Several mass vaccination campaigns that targeted a range of age groups were given as potential models for dengue vaccine delivery in these three countries. These include H1N1 influenza vaccination campaigns for young children (e.g., 5–18 month olds), pregnant women, and the elderly; and rubella elimination campaigns that involved routine immunization for one year olds, follow-up vaccination for pre-schoolers, catch-up campaigns for 1–14 year olds, and one-time “speed-up campaigns” for adolescents and adults.

#### Financing for dengue vaccination

There was general agreement among informants in Vietnam and Nicaragua – both GAVI-eligible countries – that GAVI or other donor financing would be critical for the widespread or early introduction of a dengue vaccine through the public sector. In Sri Lanka, which is now graduating from GAVI support, not all agreed that donor funding would be necessary for introduction of the vaccine into the national immunization program, given the priority of dengue within the government.

Dengue vaccine introduction into the national immunization program will be financed by the federal government in Thailand, Brazil, Mexico and Colombia – all middle-income countries that are self-financing for vaccines. However, several informants in these countries suggested that free dengue vaccination through the national program could be limited to certain age groups (e.g., children under a certain age), while older children and/or adults would have to get vaccinated in the private sector and be asked to pay. In the case of Thailand, Colombia, and Mexico, these costs could potentially be covered, at least partially, by national health insurance, social insurance or private insurance.

#### Source of dengue vaccines: local production vs. importing

Five of the eight countries in the study – India, Thailand, Vietnam, Brazil and Mexico – produce vaccines locally, and dengue vaccines are currently in development in India, Vietnam and Brazil, including the U.S. NIH chimeric vaccine licensed to producers in all three countries.

According to several interviewees in Brazil, the government would be willing to buy imported vaccine if locally-produced dengue vaccine is not yet available, although probably only on a small scale, since a high price is assumed. Thus, broad public sector use of dengue vaccines in Brazil may have to wait until a locally-produced vaccine is available in sufficient quantities. In India, a dengue vaccine used by the UIP would likely have to be produced locally or at least filled-finished in the country.

In Vietnam, according to informants, the likely steps that the government would follow would be to initially import dengue vaccine to avoid long delays in its use, then to fill-finish imported bulk vaccine locally, and finally to produce the vaccine from scratch through technology transfer. In Mexico, the possibility of the government supplier, BIRMEX, fill-finishing imported bulk vaccine was also raised. Otherwise, the company, which is the sole supplier of vaccines to the MOH, would purchase the imported finished product directly. Local production of dengue vaccines in Thailand was considered by most informants to be unlikely in the foreseeable future.

### Data required to inform decision-making about dengue vaccine introduction

While all countries will require evidence of the safety and efficacy of any new vaccine before it can be introduced into the national immunization program, both Sri Lanka and Vietnam now require that safety and immunogenicity be demonstrated in the local population through a small Phase I/II study, even for vaccines pre-qualified by WHO. (An exception is made in Vietnam for vaccines supplied by the GAVI Alliance. Immunogenicity studies are also not required in Vietnam for vaccines that have been licensed in other countries for five years or more.) Health policymakers in Sri Lanka, Thailand and Mexico expressed strong interest in conducting Phase IV post-marketing surveillance (PMS) studies once the vaccine has been introduced into the national immunization program to further monitor its safety. A vaccine researcher in Thailand also recommended using PMS to track the effects of vaccine introduction on individuals previously exposed to dengue, while Indian health officials expressed interest in studying the vaccine's tolerability in HIV positive individuals.

Several countries would require additional evidence of disease burden from different parts of the country before making a decision to introduce a dengue vaccine. Additional surveillance data would help to strengthen the case made to policymakers for dengue vaccination, and in countries, such as India, Colombia and Mexico, where targeted vaccination is likely, it would help determine which areas to prioritize for vaccination. The need for additional disease burden data for decision-making was mentioned less often in countries confident in their surveillance and reporting systems or where nation-wide vaccine introduction is assumed (e.g., Sri Lanka and Thailand).

Economic data were mentioned as critical evidence needed for policy decisions in all countries. According to health officials in India, cost-effectiveness estimates are increasingly important to the government, especially for newer, more expensive vaccines and “niche” vaccines. In Vietnam, informants reported that the Finance Ministry will only approve financing for imported vaccines if they are determined to be cost-effective. Also viewed as critical in various countries – both as stand-alone evidence and as data needed for the cost-effectiveness analyses – were data on the cost of the disease in their country, including the cost of treatment and hospitalization, the cost of vector control, and the economic impact of the illness on the poor.

## Discussion

### Summary of findings and implications

The views of policymakers and other stakeholders concerning dengue and dengue vaccines were first surveyed in 2002 in a study of four Southeast Asian countries (Cambodia, Indonesia, Philippines and Vietnam) [14]. This new survey of eight countries in both Asia and Latin America confirms the generally high level of importance accorded to dengue by government policymakers and other stakeholders across dengue-endemic countries that was found in the 2002 study, as well as strong interest in dengue vaccines for public health use. There were few clear distinctions found in the views about dengue and dengue vaccines between Asian stakeholders and those in the Americas. One of the only discernible differences was the greater interest in vaccinating adults in the Americas compared to Asia, due to the older age distribution of disease in the Americas.

As in the 2002 survey, the perceived importance of the disease and the perceived need to have more effective tools to control it are driven by the fact that dengue is increasing in incidence and spreading within countries. Adding to the sense of urgency since the 2002 study is the fact that major outbreaks are becoming an annual occurrence in several Asian countries and thus, the disease is transforming from an epidemic to an endemic or even hyper-endemic disease. While dengue appears to be worsening in magnitude and severity, substantial progress has been made in recent years in controlling such high priority diseases as tuberculosis, malaria and measles in many dengue-endemic countries, effectively increasing the relative importance of dengue.

Another key factor contributing to the sense of priority of dengue and interest in dengue vaccines is the highly visible nature of the disease. A large part of this visibility is due to the fact that it often occurs in epidemics – which attract media attention, stoke fear and even panic in the public, can overwhelm hospitals, and put a strain on municipal budgets. This is in contrast to non-epidemic diseases, such as diarrheal disease and pneumonia, which exact a higher toll than dengue in terms of morbidity and mortality in many countries, but which attract less public or media attention. The importance of an epidemic disease pattern in creating a demand for a vaccine is demonstrated by the high priority that governments in the meningitis belt of Africa placed on the development of an effective vaccine against meningococcal meningitis A – which is now being used in mass campaigns in several countries – despite the relatively low death toll from the disease [15], [16]. Dengue's visibility is further enhanced by its occurrence in cities – which are the centers of the media and political leadership – and the fact that it strikes all social classes and not just the poor.

These factors have created considerable political pressure on governments to control the disease, and according to informants, will create pressure to introduce a dengue vaccine once one is available. Recent studies into factors influencing government adoption of vaccines suggest that political pressure – often fueled by public fear or anxiety about a disease – has contributed to decisions to introduce certain new vaccines, even in the absence of solid disease burden or cost-effectiveness data [17]–[19]. Political considerations were paramount in the rapid introduction of HPV vaccines in seven developed countries, despite uncertainty about the vaccines' long-term effectiveness and the lack of country-specific cost-effectiveness data in four of the countries [18]. In some countries, decisions to fund the HPV vaccine bypassed the normal decision-making process and were even made in the context of current or upcoming elections. And in the Netherlands, the government decided to introduce meningococcal C vaccine without a favorable cost-effectiveness analysis, in part to assuage public anxiety about the disease [19]. While these examples are from developed countries, this survey strongly suggests that similar pressures will be applied to dengue vaccines in many dengue-endemic developing countries.

The findings of this and the 2002 survey also highlight the differences in how policymakers and opinion leaders in many endemic countries define disease burden as compared to global institutions and donors. While international organizations tend to define the burden of disease in terms of mortality and morbidity, and thus consider dengue a low-mortality disease of low priority, many of those dealing with the disease in endemic countries take a much more comprehensive view of the dengue disease burden. They also take into consideration the economic costs of outbreaks on health systems, the cost of vector control, as well as such immeasurable, more “political” variables as the panic that outbreaks can cause among the public; the fear among doctors of patients deteriorating rapidly due to the unpredictable nature of the disease; and the demand from parents, the media and society at large for the government to prevent and control the disease. Since country-level policymakers must consider all of these factors, they tend to accord a higher priority to dengue than do global institutions at present.

### Research activities and other factors that will facilitate the introduction of dengue vaccines

The findings of this survey provide a blueprint for research and other activities that are needed to accelerate dengue vaccine introduction in public sector immunization programs in endemic countries. Primary among the research needs are more systematic assessments of the local dengue disease burden (e.g., sentinel site surveillance). Many countries will also require locally-generated data on the cost of dengue, including cost-of-illness and vector control costs, and on the cost-effectiveness of vaccination. Studies to demonstrate the safety and immunogenicity of the vaccine in the local population will also need to be undertaken in countries with an explicit policy requiring such studies for new vaccines (e.g., Vietnam and Sri Lanka), and perhaps in other countries as well, given the unique safety concerns about dengue vaccines. Pilot introduction or vaccine demonstration projects may be the preferred route to making a decision about vaccine introduction, as mentioned by informants in several countries and as shown in the literature to be an important factor in the introduction of hepatitis B vaccine in some early adopter countries (e.g., Thailand, Taiwan, Indonesia) [17].

The development of international recommendations, such as by the WHO Strategic Advisory Group of Experts (SAGE) on immunization, could also accelerate dengue vaccine introduction. Not only was this mentioned as an important factor in several countries in the study, the history of the introduction of Haemophilus influenzae Type b (Hib) vaccine suggests that the development of stronger recommendations by WHO in 2006 calling for universal use of the vaccine was a contributing factor to the rapid adoption of the vaccine, along with intensive advocacy and GAVI support in the years following the new recommendations [20].

Three additional conditions or criteria could be critical to the introduction of dengue vaccines in developing countries. One is vaccine prices that countries consider “affordable”. Dengue vaccine introduction will likely be aided in the Americas if the vaccine can be purchased through the PAHO Revolving Fund, which obtains lower prices than countries can generally obtain on their own. Second, donor financing, especially through the GAVI Alliance, will be critical to avoid long delays in vaccine introduction in many low- and lower-middle income endemic countries. GAVI eligibility shortened the time for countries to decide to introduce Hib vaccine by 63% and made up for differences in income levels between countries [21]. Finally, according to informants in several countries, policymakers will be more inclined to introduce a dengue vaccine if it can be incorporated into the countries' childhood immunization schedules.

### Study limitations

There are a number of limitations of this study that must be considered. First, the countries included in the study were a convenience sample and not necessarily representative of all dengue-endemic countries or of all potential early adopters of dengue vaccines. Since this study represents only a subset of potential early adopters of the vaccine, additional studies in other countries would be of value. Nonetheless, the combined population of these eight countries makes up a significant portion of the global population at risk for dengue, including the largest country in each region with a substantial dengue burden (India and Brazil). Secondly, the sample of informants may not have included all major stakeholders in each country or have been representative of all stakeholders, since the sample size per country was quite small and certain key sectors, such as finance ministries, were often not available for interviews. In addition, by the time dengue vaccines become available, many of the major decision-makers and opinion leaders may have changed. Nonetheless, those interviewed in each country included representatives of groups found in the literature to be highly influential in the adoption of new vaccines, including senior Ministry of Health officials, NITAG members, officials from medical professional societies, leading academicians, and local vaccine producers [17], [22], [23].

There is also the possibility that informants' responses were biased towards playing up the importance of dengue and interest in dengue vaccines, since they were aware that the survey was being conducted by a dengue vaccine project. However, a number of respondents expressed less concern about dengue or were hesitant to embrace dengue vaccines, suggesting an atmosphere of free expression. As with all qualitative studies with open-ended responses, there is also the possibility of misunderstanding or biased interpretation of informant's responses. The structure of the interviews, which allowed for probing and clarification of responses, was designed to minimize misinterpretation. Bias could also arise in the selection of responses to report in the paper, although efforts were made in the analysis to find and examine opposing views within a country. However, many responses, especially those concerning the importance of dengue and interest in a vaccine, occurred repeatedly across respondents and across countries and are thus likely to transcend these possible biases and reflect the prevailing views of stakeholders concerning dengue and dengue vaccines in these eight countries.
